# Antidepressant‐Like Effects of Electroacupuncture by Regulating NLRP3‐mediated Hippocampal Inflammation and Pyroptosis in Rats With Post‐Stroke Depression

**DOI:** 10.1002/brb3.70670

**Published:** 2025-07-07

**Authors:** Wa Cai, Xi‐Fang Wei, Larissa Tao, Wei‐Dong Shen

**Affiliations:** ^1^ Department of Acupuncture Shanghai Shuguang Hospital Affiliated to Shanghai University of Traditional Chinese Medicine Shanghai China; ^2^ Department of Acupuncture The First Affiliated Hospital of Henan University of Chinese Medicine Zhengzhou Henan Province China

**Keywords:** electroacupuncture, hippocampal inflammation, NLRP3, pyroptosis, PSD

## Abstract

**Objective:**

This study aimed to investigate whether the antidepressant‐like effects of electroacupuncture (EA) are associated with the regulation of inflammation and pyroptosis mediated by the NLRP3 inflammasome, as well as the protection of neuron cells in the hippocampus (HP) in a rat model of post‐stroke depression (PSD).

**Methods:**

Depressive‐like behaviors (DLBs) in PSD rats were evaluated through a series of behavioral tests. The expression levels of NLRP3, caspase‐1 (Casp‐1), and apoptosis‐associated speck‐like protein (ASC), as well as the cleavage product gasdermin D (GSDMD), were assessed using real‐time PCR (RT‐PCR), immunofluorescence, and Western blot (WB). IL‐1β and IL‐18 levels were measured by RT‐PCR and WB. To upregulate NLRP3 expression, adeno‐associated virus (AAV) was injected into the hippocampus. Nissl staining was employed to evaluate neuronal morphology and count in the hippocampus.

**Results:**

EA significantly alleviated DLBs in PSD rats and suppressed the overexpression of NLRP3, Casp‐1, ASC, GSDMD, IL‐1β, and IL‐18 in the ischemic hippocampus. NLRP3 overexpression attenuated the therapeutic effects of EA on both behavioral outcomes and neuroinflammatory markers.

**Conclusions:**

EA mitigated hippocampal inflammation and pyroptosis in PSD by downregulating NLRP3 inflammasome activation, indicating that NLRP3 may serve as a potential therapeutic target for PSD.

## Graphical abstract

Electroacupuncture was found to down‐regulate NLRP3 inflammasome activation to reduce hippocampal inflammation and pyroptosis, suggesting that NLRP3 inflammasome could be a potential target for PSD treatment.

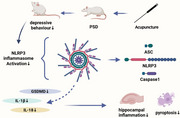

## 1 Introduction

Post‐stroke depression (PSD) is a common neuropsychiatric complication following stroke, significantly diminishing patients’ quality of life and hindering recovery efforts (Wan et al. 2024, Zheng et al. 2024). The incidence of depression markedly increases within the first few weeks to three months after stroke, adversely affecting rehabilitation outcomes and reducing long‐term survival rates (Gu et al. 2024, Liu et al. 2023, Masuccio et al. 2024). Although pharmacotherapies, such as selective serotonin reuptake inhibitors, are widely used and often effective, they are associated with notable side effects, including myocardial infarction, sexual dysfunction, and an increased risk of hemorrhage (Coupland et al. 2011, Hackett et al. 2008). These limitations highlight the urgent need for safer and more tolerable therapeutic alternatives for PSD.

Recent studies have underscored the critical role of hippocampal (HP) inflammation in the pathogenesis of both stroke (Zhou et al. 2022) and depression (Ruilian et al. 2021, Xu et al. 2016). A meta‐analysis has concluded that proinflammatory cytokines are significantly linked to the development of PSD (Chen et al. 2020). Experimental research has further demonstrated that inhibition of HP inflammation can ameliorate depressive‐like behaviors (DLBs) in PSD animal models (Li et al. 2017). Clinically, stroke patients with higher serum proinflammatory cytokine levels contribute to a higher PSD risk (Kang et al. 2016, Kim et al. 2017), suggesting that hippocampal inflammatory activation is a key driver of post‐stroke mood disturbances.

The NLRP3 (NOD‐, LRR‐, and pyrin domain‐containing protein 3) inflammasome is a central regulator of inflammation, comprising NLRP3, the adaptor protein ASC (apoptosis‐associated speck‐like protein containing a CARD), and caspase‐1 (Casp‐1) (Zhong et al. 2018). Upon activation, NLRP3 promotes the cleavage of pro‐Casp‐1 into its active form, which in turn cleaves gasdermin D (GSDMD), facilitating the maturation and release of interleukins IL‐1β and IL‐18. This process leads to pyroptosis, a proinflammatory form of programmed cell death (McKenzie et al. 2018, Fann et al. 2018, Ren et al. 2018). The resulting inflammatory cascade contributes to neuronal damage and cognitive impairment, which are believed to exacerbate depressive symptoms (You et al. 2011, Czeh and Nagy 2018).

Acupuncture has emerged as a promising alternative for PSD, particularly for patients who are unable to tolerate conventional antidepressants (Liu et al. 2021, Wang et al. 2021). Electroacupuncture (EA), a modern adaptation of traditional acupuncture, has shown efficacy in alleviating DLBs by suppressing hippocampal inflammation in animal models (Chen et al. 2022, Wang et al. 2022, Zhou et al. 2022). Additionally, EA has been shown to reduce neuronal apoptosis in the hippocampus (HP) (Cheng et al. 2021) and promote neuroplasticity by enhancing dendritic arborization and spine density in the CA1 region of the hippocampus (Davila‐Hernandez et al. 2018).

Given these findings, our study aimed to investigate whether the antidepressant effects of EA in PSD rats are mediated by the inhibition of NLRP3 inflammasome activation, suppression of pyroptosis, and protection of hippocampal neurons.

## 2 Methods

### 2.1 Animals

Male Sprague–Dawley rats (2 months, 180–200 g) were obtained from Beijing Wei Tong Li Hua Laboratory Animal Technology Co., Ltd. (Shanghai, China). Animals were housed under standard laboratory conditions (22–24°C, 55%‐65% humidity, 12‐h light‐dark cycle) with free access to food and water. All experimental procedures were approved by the Ethics Committee of Shanghai University of Traditional Chinese Medicine (No. PZOHUTCM210305007) and conducted in accordance with the ARRIVE guidelines, the Animals (Scientific Procedures) Act 1986, and EU Directive 2010/63 on the protection of animals utilized for scientific purposes.

After a 1‐week acclimatization period, 60 rats were randomly divided into two groups: sham (*n* = 8) and middle cerebral artery occlusion (MCAO, *n* = 52). Of the 52 rats undergoing MCAO surgery, 12 died within 72 h post‐surgery. The remaining 40 surviving rats were randomly assigned to five groups (*n* = 8/group): PSD, EA, PSD + overexpression (OE)‐NLRP3 + EA, PSD + null virus (NV) + EA, and PSD + OE‐NLRP3.

### 2.2 Establishment of the PSD Model

The PSD model was developed by middle cerebral artery occlusion (MCAO) plus chronic unpredictable mild stress (CUMS) (Fan et al. 2023, Hu et al. 2019, Jiang et al. 2021, Liu et al. 2024, Lv et al. 2024). MCAO was conducted to generate cerebral ischemia (Belayev et al. 1996). Under sodium pentobarbital anesthesia, a midline cervical incision was made to expose the right common carotid artery (CCA), external carotid artery (ECA), and internal carotid artery (ICA). A 0.32 mm‐diameter nylon monofilament (Beijing Xinong Technology Co. Ltd., China) was inserted into the ICA to occlude the middle cerebral artery. After 90 minutes of ischemia, the filament was removed to allow reperfusion.

Twenty‐four hours after surgery, neurological function was assessed using the Longa‐Z scoring method (Longa et al. 1989). Rats with scores between 1 and 3 were included in the study. Sham‐operated rats underwent the same surgical procedure without filament insertion.

Following a 7‐day postoperative recovery period, all groups—except the sham group—were subjected to a 6‐week CUMS protocol (Willner et al. 1992). The CUMS procedure involved daily exposure to any two randomly selected stressors from the following set: 24‐h food or water deprivation, overnight illumination, soiled bedding (24 h), 45° cage tilt (24 h), 4‐h restraint, 2‐h restraint at 4°C, 8‐h noise exposure, 6‐hour strobe lighting, and 15‐min cage shaking.

### 2.3 EA Stimulus

**FIGURE 1:**
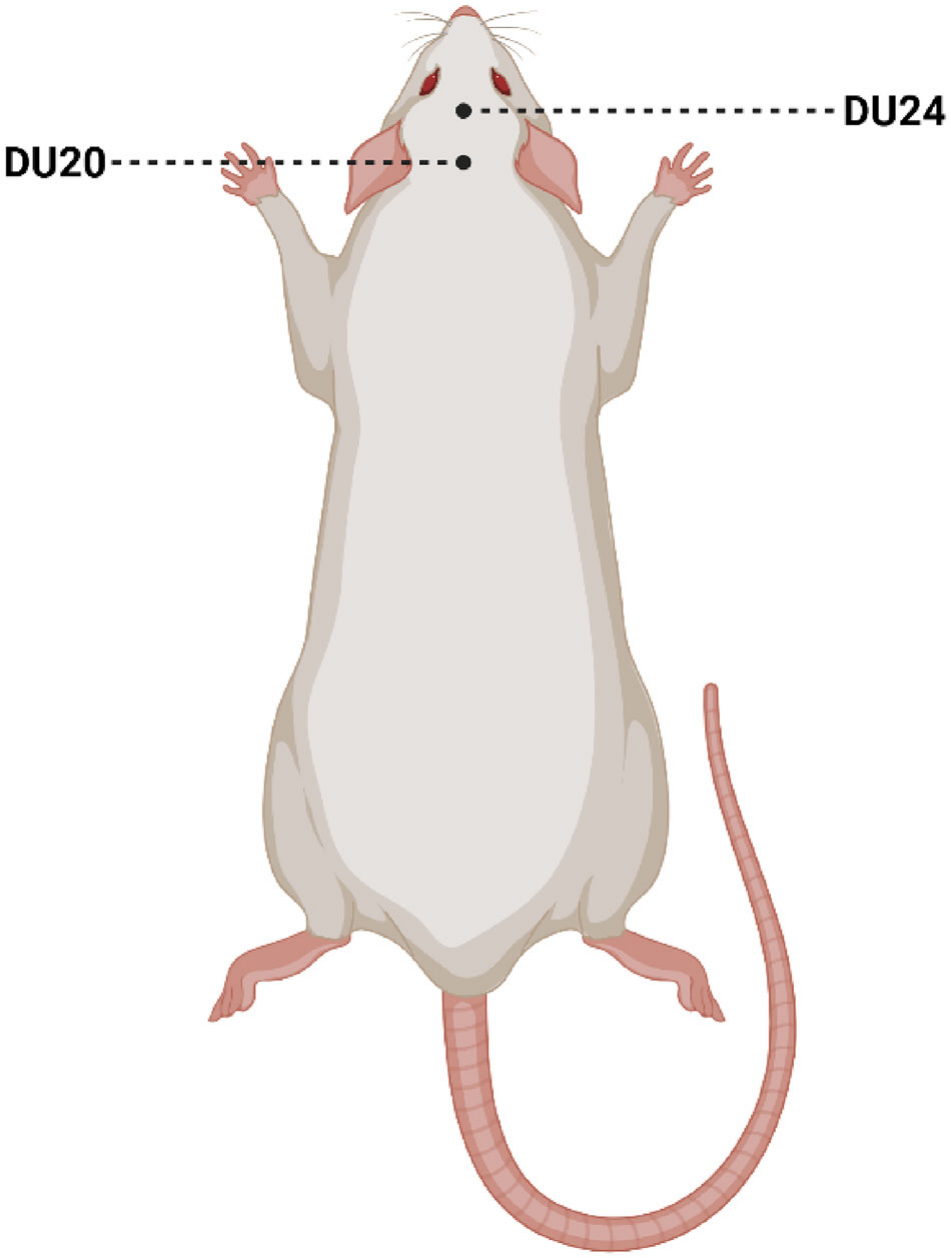
Acupoint locations of DU20 and DU24 on the animal.

EA was administered at the Baihui (DU20) and Shenting (DU24) acupoints (Guo 2021) (Figure 1). Stainless steel needles (0.25 mm diameter, 40 mm length; Jiajian Medical Supplies Co. Ltd., Jiangsu, China) were inserted to a depth of 5–8 mm. Acupoint selection was based on our previous studies, which demonstrated significant efficacy in alleviating depressive‐like symptoms in animal models (Cai et al. 2019, Cai et al. 2023, Cai et al. 2024). The inserted needles were connected to an electroacupuncture device (Hwato SDZ‐II, Suzhou Medical Supplies Co. Ltd., Jiangsu, China), delivering continuous stimulation at a frequency of 20 Hz and an intensity of 1 mA. Each session lasted 20 min. EA treatment began at the end of the third week of the CUMS protocol and was administered once daily for three consecutive weeks.

### 2.4 Behavioral Tests

All behavioral assessments were conducted on day 21 and day 42 (before and after EA treatment) of the experiment:

#### 2.4.1 Sucrose Preference Test (SPT)

The SPT was used to assess anhedonia in rats (Tang et al. 2015). On day 1, rats were provided with two bottles of 1% sucrose solution for 24 h. On day 2, one bottle of 1% sucrose and one bottle of purified water were presented, with bottle positions switched after 12 h to avoid side bias. On day 3, rats underwent 24 h of water deprivation. On day 4, rats were given access to both sucrose and water bottles for 2 h. Sucrose preference was calculated as sucrose intake (g) / [sucrose intake (g) + water intake (g)].

#### 2.4.2 Open Field Test (OFT)

The OFT was used to evaluate locomotor activity and exploratory behavior (Tang et al. 2015). Each rat was placed in the center of a dark, open‐field box (100 × 100 × 50 cm) equipped with an automated data acquisition system (Xinruan Information & Technology Co., Ltd., Shanghai, China). Rats were allowed to explore freely for 5 min. The total distance traveled, number of rearing events, and number of entries into the central area were recorded. The arena was thoroughly cleaned between sessions to eliminate olfactory cues.

#### 2.4.3 Forced Swim Test (FST)

The FST was conducted to assess behavioral despair. Each rat was placed in a transparent cylindrical tank (height: 55 cm, diameter: 22 cm) filled with water (20–22°C) to a depth of approximately 30 cm. The test lasted for 6 min, with immobility time recorded during the final 4 min. Immobility was defined as the absence of active swimming, with minimal movements required only to keep the head above water (Detke and Lucki 1996).

### 2.5 Viral Injection

Two weeks after MCAO surgery, rats were anesthetized with sodium pentobarbital and secured in a stereotaxic apparatus for hippocampal injection. Recombinant adeno‐associated virus (AAV) vectors were injected into the hippocampus at a rate of 100 nL/min, with a total volume of 1000 nL. Stereotaxic coordinates were as follows: anteroposterior (AP) −3.4 mm, mediolateral (ML) + 4.0 mm, and dorsoventral (DV) −3.5 mm. To induce NLRP3 overexpression, we used recombinant AAV encoding NLRP3 (pAAV‐CMV‐Nlrp3‐3FLAG). Control rats received AAV encoding a null vector (pAAV‐CMV‐MCS‐3FLAG; all from Obio Technology, Shanghai, China).

### 2.6 Brain Sample Collection

At the end of the experiment, rats were deeply anesthetized with an overdose of anesthesia using 2 mL of sodium pentobarbital injected intraperitoneally. For qPCR and Western blot (WB) analyses, the brains were rapidly removed, and hippocampal (HP) tissues from the ischemic side were dissected, flash‐frozen in liquid nitrogen, and stored at −80°C until usage. For immunofluorescence and Nissl staining, rats were transcardially perfused with 4% paraformaldehyde. Brains were post‐fixed, dehydrated, embedded in optimal cutting temperature (OCT) compound (Tissue‐Tek), and sectioned at 30 µm using a freezing microtome (Leica CM1950, Nussloch, Germany). Serial sections were collected into six sets and maintained in 50% glycerol at −20°C.

### 2.7 Quantitative Real‐Time (qRT) PCR

Gene expression in the HP ischemic penumbra was analyzed by qRT‐PCR. Total RNA was extracted via TRIzol reagent (Invitrogen, Thermo Fisher Scientific, Massachusetts, USA), and cDNA was synthesized following the protocols with a cDNA Synthesis Kit (Wuhan Servicebio Technology, Wuhan, China). qPCR was carried out through a thermocycler (Bio‐Rad Laboratories, California, USA). The specific primers used for amplification are listed below (Table 1).

**TABLE 1:** The specific primers used for amplification in qRT‐PCR.

Target	Sequence
R‐NLRP3‐S	GATTTCTCCACAACTCACCCAA
R‐NLRP3‐A	AGTCTGGAAGAACAGGCAACAT
R‐ASC‐S	ACTATCTGGAGGGGTATGGCTT
R‐ASC‐A	CAATGAGTGCTTGCCTGTGTT
R‐Caspase1‐S	AAGCCCAAGGTTATCATTATTCAGG
R‐Caspase1‐A	ATCCCTCTTCGGAGTTCCCTAC
R‐GSDMD‐S	CAGGCAGCATCCTTGAGTGTC
R‐GSDMD‐A	CCAAGACGTGCTTCACCAACT
R‐IL‐18‐S	AACAGCCAACGAATCCCAGAC
R‐IL‐18‐A	TTGTTTTTACAGGAGAGGGTAGACA
R‐IL‐1β‐S	TGTGACTCGTGGGATGATGAC
R‐IL‐1β‐A	CCACTTGTTGGCTTATGTTCTGTC

### 2.8 Western Blot

Total protein extraction from the ischemic HP was carried out by RIPA tissue lysis buffer. Protein concentrations were determined with a Bicarbonate Protein Assay Kit (Wuhan Servicebio Technology, Wuhan, China). The WB was performed by incubating the membranes with primary antibodies: anti‐NLRP3 (1:1000, NOVUS), ASC (1:500, Santa Cruz), Casp‐1 (1:1000, Proteintech), GSDMD (1:500, Santa Cruz), and IL‐1β/18 (1:1000, Abcam) at 4°C for a whole night, followed by secondary antibody incubation.

### 2.9 Immunofluorescent Staining

Coronal sections of the ischemic HP were prepared at a thickness of 12 µm. For immunofluorescence, we deployed the following primary antibodies: anti‐NLRP3 (1:1000, NOVUS), ASC (1:500, Santa Cruz), Casp‐1 (1:1000, Proteintech), NeuN (1:1000, Abcam), and GSDMD (1:500, Santa Cruz). Fluorescence‐labeled secondary antibodies were applied, counterstaining nuclei with DAPI (Wuhan Servicebio Technology, Wuhan, China). Visualization was performed using an ECLIPSE C1 upright fluorescence microscope (Nikon Instruments Inc., Shanghai, China), and mean fluorescence intensity was quantified by ImageJ software (v1.51, National Institutes of Health, Bethesda, USA).

### 2.10 Nissl Staining

Ischemic HP sections were subjected to staining with 0.5% thionin at room temperature, dehydrating in 95% ethanol, baking at 65°C, clearing with xylene, and sealing with neutral gum. Ischemic HP region images were captured using a light microscope (Nikon Eclipse E100). Neuronal counting was performed with the ImageJ software.

### 2.11 Statistical Analyses

Statistical analyses were conducted via SPSS software (v21.0; SPSS Inc., Chicago, IL, USA). Data normality was validated prior to analysis. Group differences were evaluated by one‐way ANOVA, with P < 0.05 indicating statistical significance.

## 3 Results

### 3.1 EA Ameliorated DLBs of PSD Rats

**FIGURE 2:**
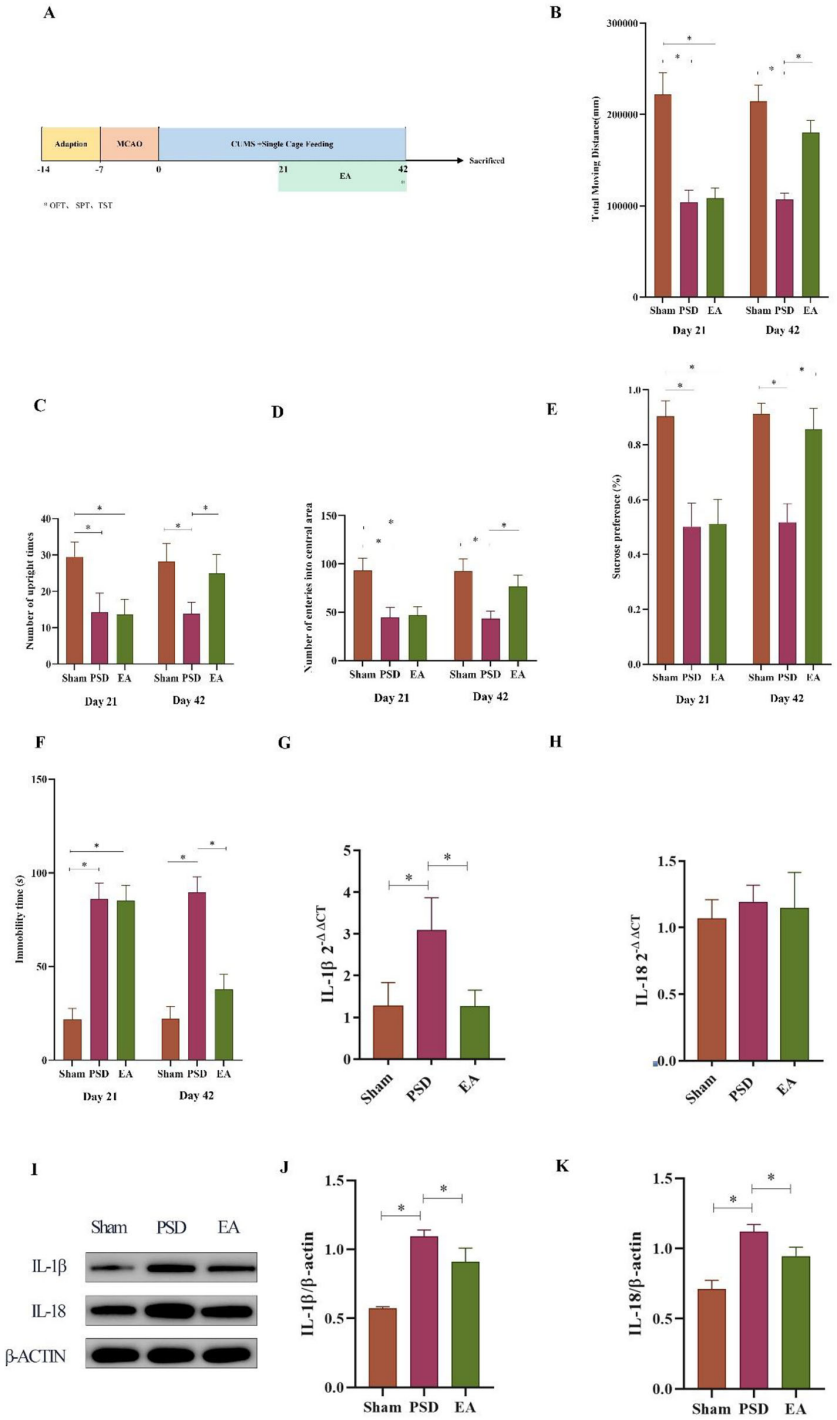
Impact of EA on the depressive‐like behaviors and inflammatory response in the ischemic hippocampus of PSD rats. **(A)** Schedule of experimental procedures. **OFT: (B)** Total moving distance, **(C)** Upright time number, and **(D)** Number of entries into the central area. **(E) SPT**: Sucrose preference ratio. **(F) FST**: Immobility time. **(G‐H)** HP mRNA and protein expressions of **(G, J)** IL‐1β and **(H, K)** IL‐18. **(I‐K)**. **Data Interpretation**: Mean ± SDs. ^*^ *p* < 0.05. One‐way ANOVA for **B‐K**. EA, electroacupuncture; FST, forced swim test; OFT, open field test; PSD, post‐stroke depression; SPT, sucrose preference test.

Rats in the PSD model group exhibited marked depressive‐like behaviors, as indicated by significant reductions in total distance traveled, rearing frequency, central zone entries in the open field test, and sucrose preference in the SPT, along with increased immobility time in the forced swim test. EA treatment significantly improved these behavioral impairments. Specifically, EA increased locomotor and exploratory activity, enhanced sucrose preference, and reduced immobility time compared to the untreated PSD group (Figures 2A **‐F**).

### 3.2 EA Inhibited Inflammatory Response in the Ischemic HP

RT‐PCR analysis showed a significant elevation in IL‐1β mRNA expression in the ischemic hippocampus of PSD rats compared to the sham group. EA treatment markedly reduced this upregulation. Although IL‐18 mRNA levels were also elevated in PSD rats, EA administration led to a reduction that did not reach statistical significance (Figures 2G **–H**). These mRNA findings were further supported by Western blot analysis, which demonstrated parallel trends in protein expression (Figures 2I **–K**). Collectively, these results indicate that EA mitigates the inflammatory response in the ischemic hippocampus of PSD rats.

### 3.3 EA Suppressed NLRP3 Activation in the Ischemic HP

**FIGURE 3:**
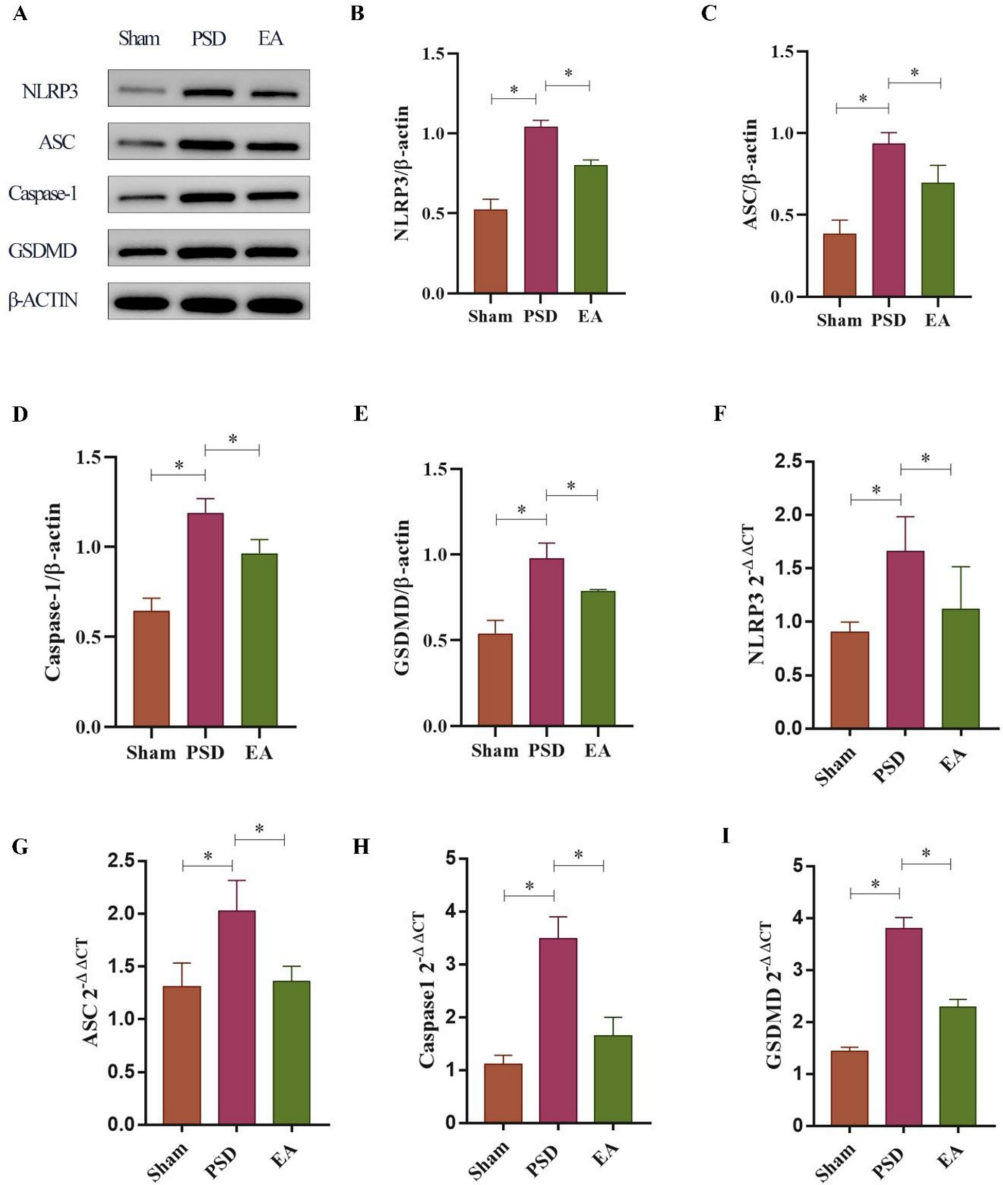
Western blot and RT PCR analysis of EA impact on NLRP3 activation in the ischemic hippocampus of PSD rats. **(A‐E)** HP protein and mRNA expressions of **(B, F)** NLRP3, **(C, G)** ASC, **(D, H)** Casp‐1, and **(E, I)** GSDMD. **Data Interpretation**: Mean ± SDs. ^*^ *p* < 0.05. One‐way ANOVA for **B‐I**. EA, electroacupuncture; PSD, post‐stroke depression.

**FIGURE 4:**
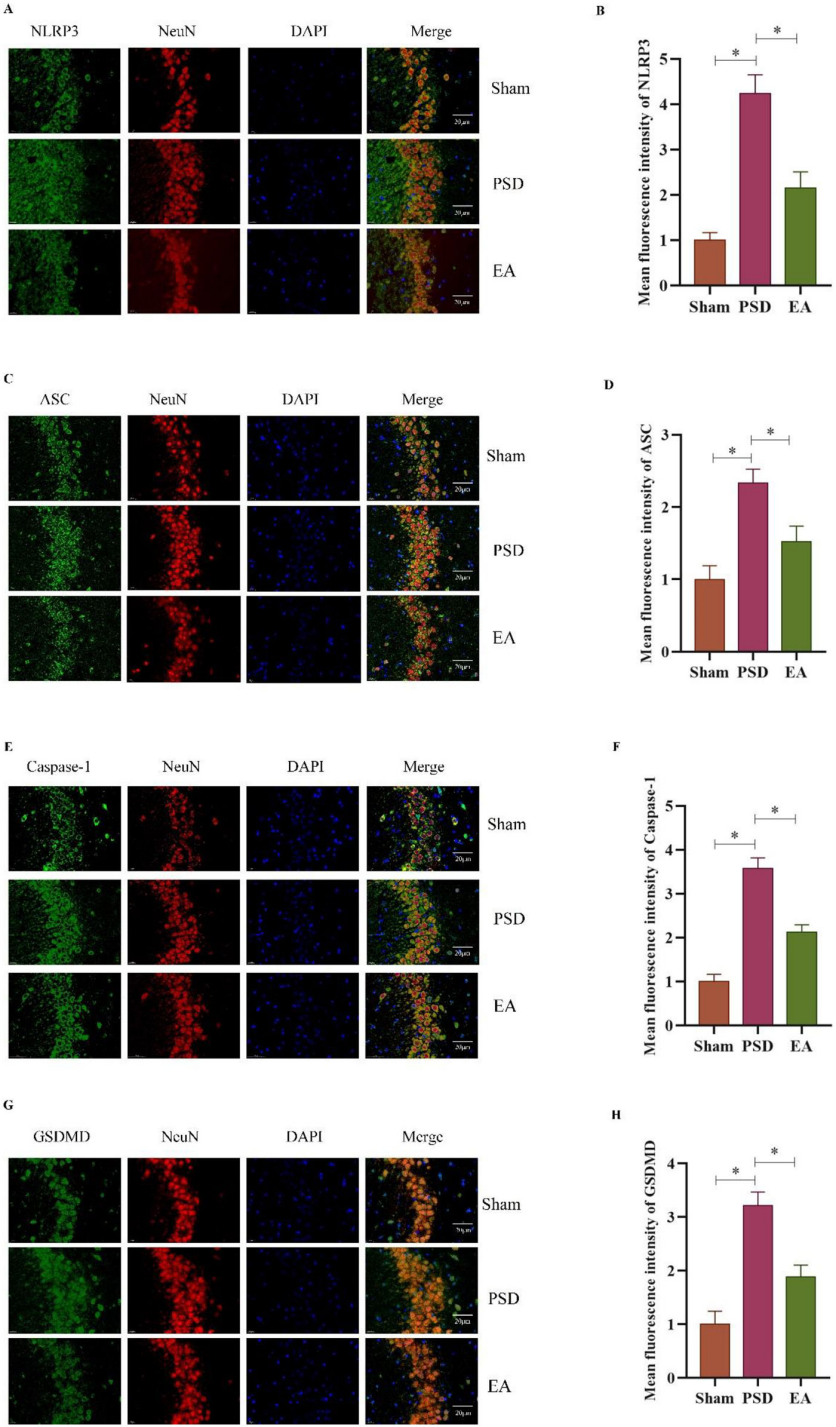
Immunofluorescence analysis of EA on NLRP3 activation in the ischemic hippocampus of PSD rats. **(A‐H)** Immunofluorescence staining expression of **(A‐B)** NLRP3, **(C‐D)** ASC, **(E‐F)** Casp‐1, and **(G‐H)** GSDMD (Scale bar = 20 µm). **Data Interpretation**: Mean ± SDs. *p < 0.05. One‐way ANOVA for **A‐H**. EA, electroacupuncture; PSD, post‐stroke depression.

Both mRNA and protein expression levels of NLRP3, ASC, Casp‐1, and GSDMD were significantly upregulated in the ischemic hippocampus of PSD rats compared to the sham group. EA treatment effectively downregulated the expression of these inflammasome‐related markers (Figures 3A **‐I**). Immunofluorescence staining further confirmed that NLRP3 was expressed in hippocampal neurons. In PSD rats, increased neural expression of NLRP3, ASC, Casp‐1, and GSDMD was observed in the CA3 region of the ischemic hippocampus. EA treatment markedly attenuated these elevations (Figures 4A **‐H**). These findings suggest that EA inhibits NLRP3 inflammasome activation at both the molecular and cellular levels in the hippocampus of PSD rats.

### 3.4 EA Reduced the Loss of Neuron Cells to Attenuate Neuronal Pyroptosis in the Ischemic HP

**FIGURE 5:**
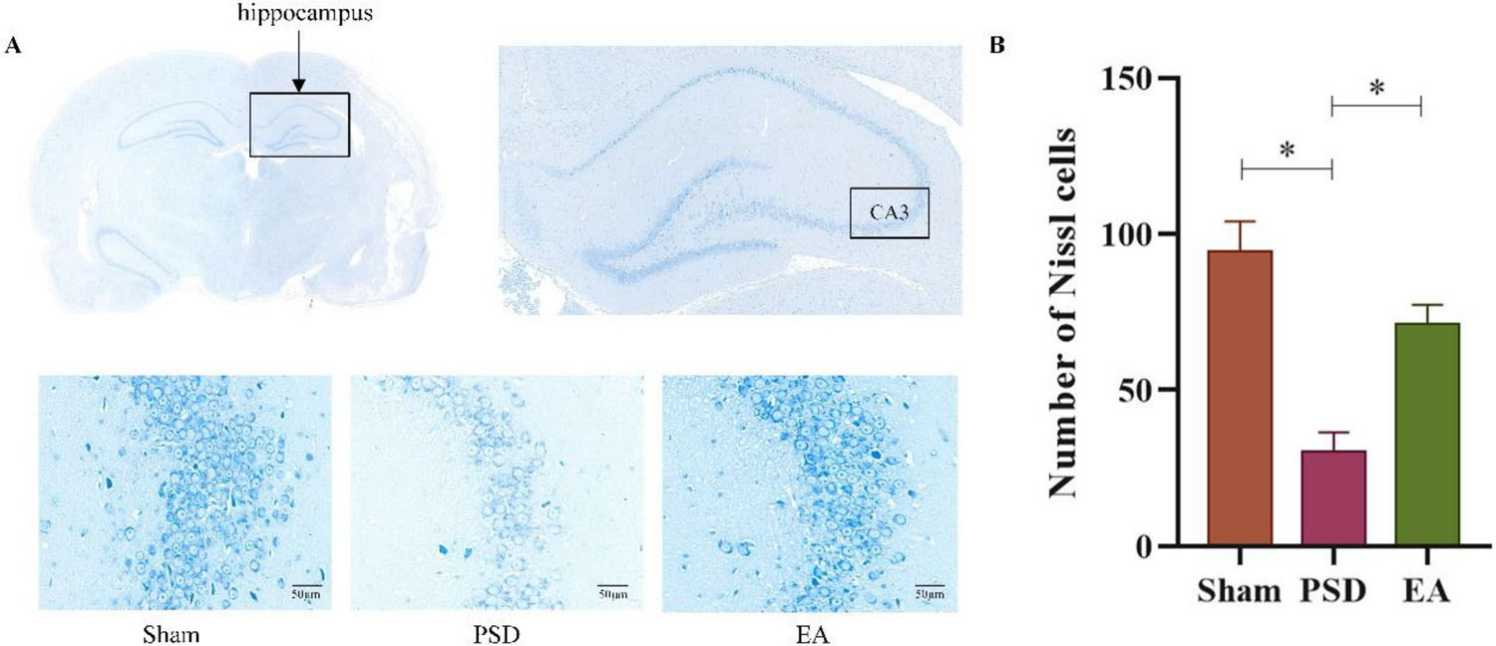
Nissl staining in the CA3 regions of hippocampus. **(A)** Nissl staining Images (Scale bar = 50 µm). **(B)** Quantitative analysis of the neuron numbers. **Data Interpretation**: Mean ± SDs. *P < 0.05. One‐way ANOVA for **B**.

Nissl staining revealed marked neuronal damage in the ischemic hippocampus of PSD rats, characterized by disorganized cell arrangement, irregular morphology, and a reduced number of Nissl‐positive neurons compared to the sham group (Figures 5A **‐B**). EA treatment significantly mitigated these pathological changes, preserving neuronal structure and reducing cell loss. These findings suggest that EA attenuates neuronal pyroptosis and protects hippocampal neurons in PSD rats.

### 3.5 Effects of EA on DLBs and HP Inflammation Response were Reversed by NLRP3 Upregulation

**FIGURE 6:**
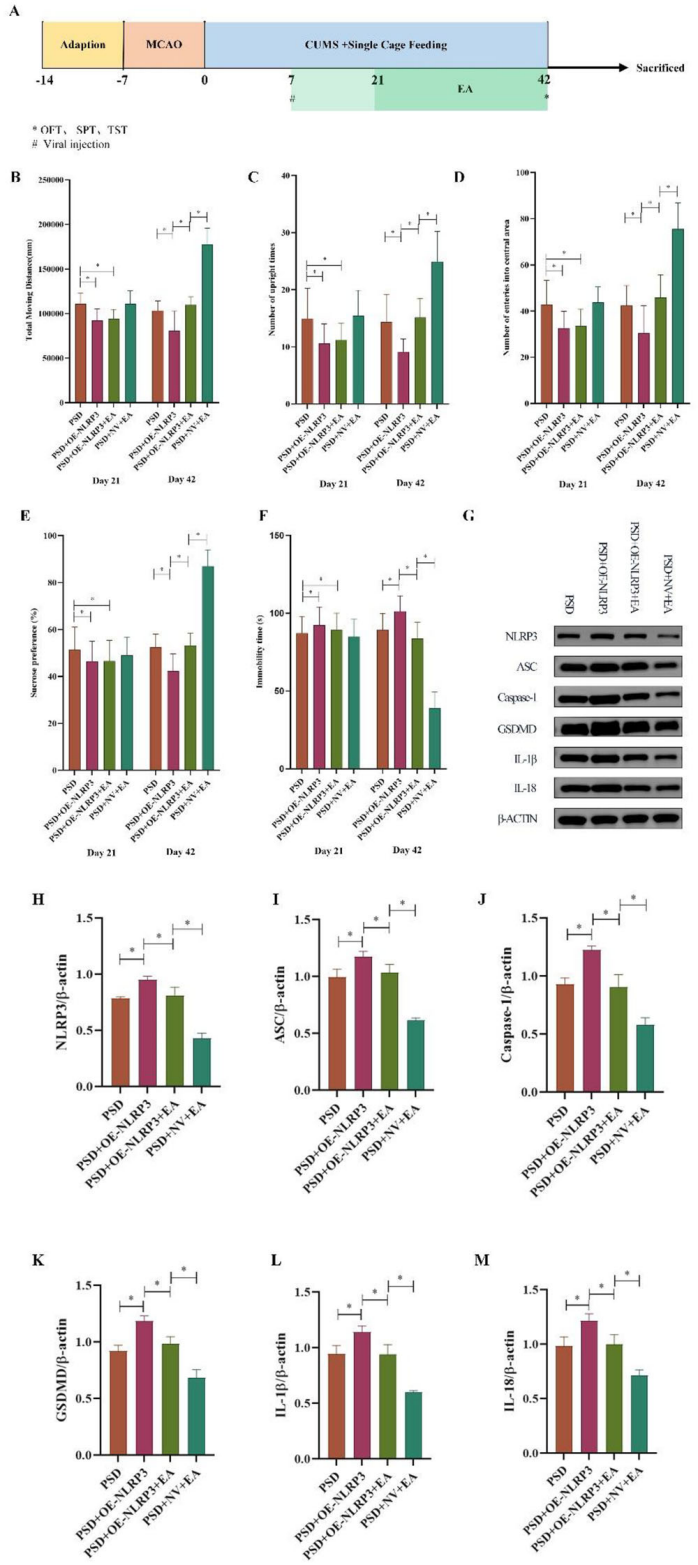
NLRP3 overexpression impeded the EA effect on depressive‐like behaviors and hippocampal inflammation response in PSD rats. **(A)** Schedule of experimental procedures. **OFT: (B)** Total moving distance, **(C)** Upright number, and **(D)** Number of entries into the central area. **(E) SPT**: Sucrose preference ratio. **(F) FST**: Immobility time. **(G‐M)** HP protein expression of **(H)** NLRP3, **(I)** ASC, **(J)** Casp‐1, **(K)** GSDMD, **(L)** IL‐1β, and **(M)** IL‐18. **Data Interpretation**: Mean ± SDs. ^*^ *P* < 0.05; One‐way ANOVA for **B‐M**. EA, electroacupuncture; FST, forced swim test; NV, null vectors; OE, overexpress; OFT, open field test; PSD, post‐stroke depression; SPT, sucrose preference test.

To validate the role of NLRP3 in mediating the antidepressant effects of EA, an AAV encoding NLRP3 (PSD + OE‐NLRP3 + EA) was microinjected into the hippocampus of PSD rats receiving EA treatment seven days post‐MCAO (Figure 6A). NLRP3 overexpression significantly increased the protein levels of NLRP3, ASC, Casp‐1, and GSDMD in the hippocampus compared to the EA‐treated PSD group without viral transduction (Figures 6G **–K**).

Behavioral assessments revealed that, in contrast to the PSD + NV + EA group, rats in the PSD + OE‐NLRP3 + EA group displayed a significant decline in total distance traveled, rearing events, central zone entries, and sucrose preference, alongside increased immobility time (Figures 6B **‐F**). These results indicate that NLRP3 overexpression effectively abolished the antidepressant‐like effects of EA.

Additionally, IL‐1β and IL‐18 protein levels were significantly elevated in the hippocampus of NLRP3‐overexpressing rats compared to the NV control group (Figures 6L **‐M**), further confirming that NLRP3 upregulation reverses the anti‐inflammatory effects of EA in PSD rats.

## 4 Discussion

**FIGURE 7:**
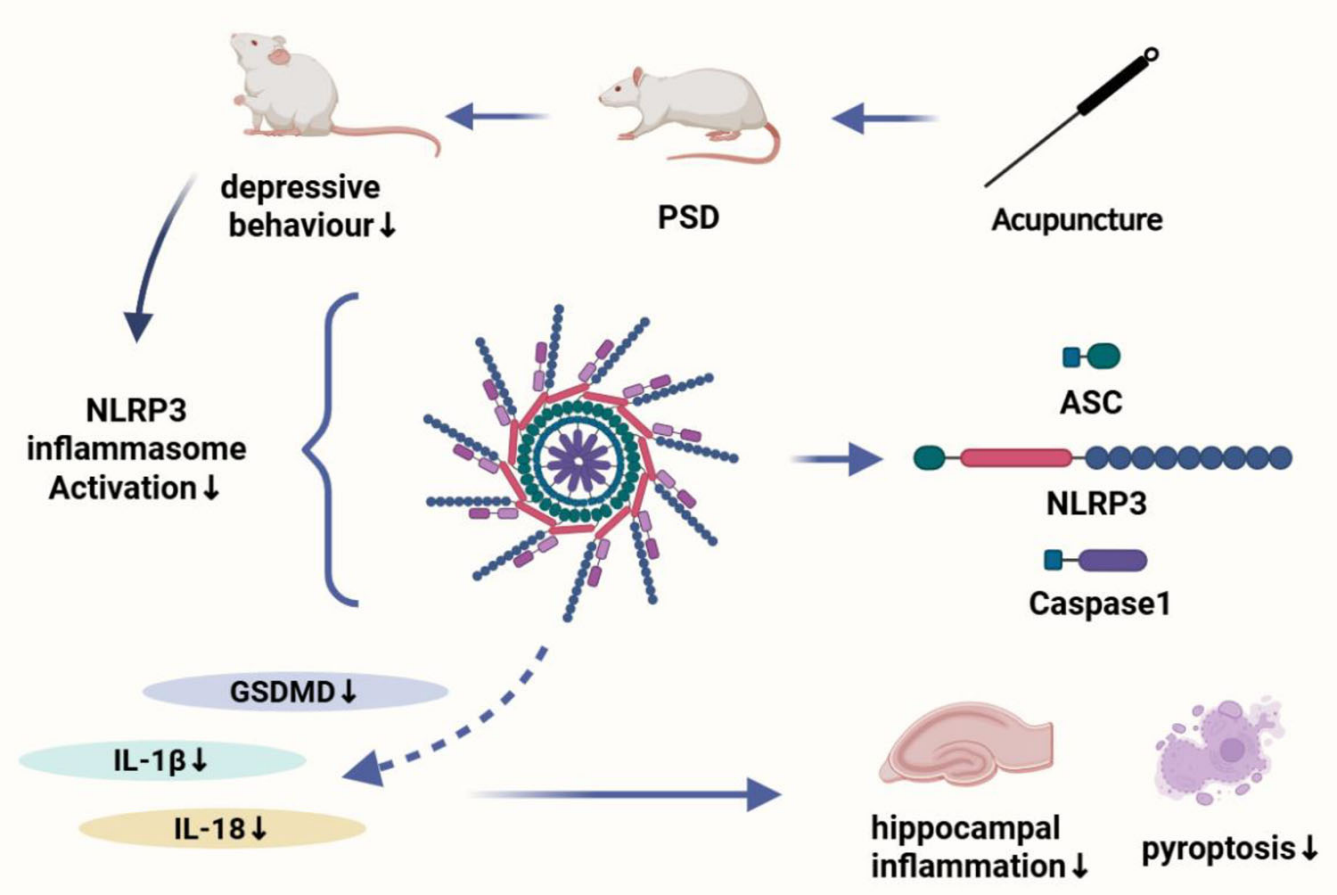
EA could alleviate depressive‐like behaviors by suppressing hippocampal inflammation response and pyroptosis by inhibiting NLRP3 activation.

This study demonstrated that EA exerts significant antidepressant effects in a PSD rat model by inhibiting NLRP3 inflammasome activation, thereby reducing hippocampal inflammation and neuronal pyroptosis. These findings suggest that NLRP3 may serve as a promising therapeutic target for PSD (**Figure** **7**).

Following ischemic stroke, oxygen and glucose deprivation lead to impaired ATP production and extensive neuronal damage. This cellular stress triggers the release of danger‐associated molecular patterns (DAMPs), which are recognized by toll‐like receptors (TLRs) (Kaur and Ling 2009). DAMP‐TLR interaction activates NF‐κB signaling, promoting IL‐1β pro‐inflammatory cytokine synthesis (Yao et al. 2013, Yao et al. 2013), thereafter processed by NLRP3 activation (Hanamsagar et al. 2011). Blocking IL‐1β signaling by hampering NLRP3 reduces brain inflammation and injury in rats after stroke (Abulafia et al. 2009, de et al. 2009, Garcia et al. 1995, Relton et al. 1996). Moreover, stroke‐triggered IL‐1β/18 and TNF‐α pro‐inflammatory cytokine upregulation can amplify neuroinflammation, activate indoleamine 2,3‐dioxygenase, and reduce serotonin levels, all of which are implicated in PSD pathophysiology (Fang and Cheng 2009, Spalletta et al. 2006). Clinically, elevated serum IL‐1β/18 levels at stroke onset are related to escalated risk of developing PSD (Kang et al. 2016, Kim et al. 2017).

The hippocampus (HP), a critical structure within the limbic system, is composed of the dentate gyrus (DG) and hippocampal gyrus (CA1–CA4 regions), with the latter rich in pyramidal neurons involved in mood regulation. Patients with depression often exhibit reduced volumes in the CA1–CA3 and DG regions (Huang et al. 2013). Although structural and functional alterations in the HP are associated with depressive disorders, the specific HP subregion most implicated in PSD remains uncertain. Notably, depression models have been reported to display significant neuronal loss in the CA3 region (Kempermann and Kronenberg 2003)—a finding corroborated by our current results, which demonstrated that EA mitigated neuronal loss in the CA3 region of PSD rat.

The hippocampal inflammation is closely associated with depression (Mokhtari et al. 2023, Mokhtari et al. 2023). Inhibition of HP inflammation has been reported to alleviate DLBs in LPS‐induced mice (Li et al. 2017). CUMS exposure can activate the NLRP3 inflammasome, promoting ASC oligomerization, caspase‐1 activation, GSDMD cleavage, and ultimately pyroptosis (Faria et al. 2021). Accumulating evidence supports the role of NLRP3‐induced inflammation and pyroptosis in depression pathogenesis (Kaufmann et al. 2017). Our study confirmed that NLRP3 contributes to hippocampal inflammation, neuronal loss, and pyroptosis in PSD rats—all of which were significantly alleviated by EA. While previous research has shown NLRP3 activation in both microglia and neurons (Gong et al. 2018), our study focused on neuronal NLRP3 expression and demonstrated that EA downregulates neuronal NLRP3 levels, aligning with recent findings (Jiang et al. 2019). Importantly, overexpression of NLRP3 in EA‐treated PSD rats reversed the behavioral and anti‐inflammatory effects of EA, further supporting the pivotal role of NLRP3 in mediating EA's therapeutic effects.

From a traditional Chinese medicine (TCM) perspective, DU20 and DU24 can efficiently alleviate depressive behaviors in PSD (Wang et al. 2021, Zhang et al. 2021). DU20, located at the vertex of the head, is believed to elevate clear yang, while DU24 calms the mind and alleviates stress. The combination of DU20 and DU24 could regulate qi and relieve depression (Liang and Wang 2021). Our previous work has shown that EA at these points improves depressive‐like behaviors in CUMS‐ and LPS‐induced models (Zhang et al. 2020, Zhang et al. 2021). The present study extended these findings to a PSD model, showing that EA at DU20 and DU24 alleviates depressive symptoms by inhibiting NLRP3‐mediated inflammation and protecting hippocampal neurons.

However, several limitations must be acknowledged. First, the relatively small sample size may limit the generalizability of the findings. Future studies should include larger cohorts. Second, needle insertion may cause discomfort or stress, potentially confounding behavioral outcomes. Third, the upstream regulators and downstream pathways of NLRP3‐induced pyroptosis were not explored and warrant further investigation. Lastly, other hippocampal subregions such as the DG and CA1–CA4 were not comprehensively assessed and should be included in future analyses.

In summary, our findings demonstrate that the antidepressant effects of EA in PSD are closely linked to its ability to inhibit NLRP3 activation, reduce hippocampal inflammation and pyroptosis, and preserve neuronal integrity. This study highlights the pathological role of hippocampal neuroinflammation in PSD and identifies NLRP3 as a potential target for intervention.

## 5 Conclusion

This study provides novel evidence that electroacupuncture exerts antidepressant effects in a PSD rat model by suppressing NLRP3 inflammasome activation, thereby reducing hippocampal inflammation and pyroptosis. These findings suggest that targeting NLRP3 may represent a promising therapeutic strategy for the treatment of post‐stroke depression.

## Author Contributions

CW and SWD: Study design. CW and WXF: Experimental procedure. WXF: Statistical analysis. CW: Manuscript drafting. LT: Manuscript revision and finalization. All authors read and authorized the final manuscript.

## Ethics Statement

The Shanghai University of Traditional Chinese Medicine Animal Research Ethics Committee authorized the experiment (PZSHUTCM210305007).

## Conflicts of Interest

The authors declare no conflicts of interest.

## Peer Review

The peer review history for this article is available at https://publons.com/publon/10.1002/brb3.70670.

## Data Availability

The data that support the findings of this study are available from the corresponding author upon reasonable request.
